# The eye-voice lead during oral reading in developmental dyslexia

**DOI:** 10.3389/fnhum.2013.00696

**Published:** 2013-11-06

**Authors:** Maria De Luca, Maria Pontillo, Silvia Primativo, Donatella Spinelli, Pierluigi Zoccolotti

**Affiliations:** ^1^Neuropsychology Unit, IRCCS Fondazione Santa LuciaRome, Italy; ^2^Department of Psychology, Sapienza University of RomeRome, Italy; ^3^Department of Human Movement Sciences and Health, University of Rome «Foro Italico»Rome, Italy

**Keywords:** reading, eye movements, eye-voice lead, dyslexia, pronunciation time

## Abstract

In reading aloud, the eye typically leads over voice position. In the present study, eye movements and voice utterances were simultaneously recorded and tracked during the reading of a meaningful text to evaluate the eye-voice lead in 16 dyslexic and 16 same-age control readers. Dyslexic children were slower than control peers in reading texts. Their slowness was characterized by a great number of silent pauses and sounding-out behaviors and a small lengthening of word articulation times. Regarding eye movements, dyslexic readers made many more eye fixations (and generally smaller rightward saccades) than controls. Eye movements and voice (which were shifted in time because of the eye-voice lead) were synchronized in dyslexic readers as well as controls. As expected, the eye-voice lead was significantly smaller in dyslexic than control readers, confirming early observations by Buswell (1921) and Fairbanks (1937). The eye-voice lead was significantly correlated with several eye movements and voice parameters, particularly number of fixations and silent pauses. The difference in performance between dyslexic and control readers across several eye and voice parameters was expressed by a ratio of about 2. We propose that referring to proportional differences allows for a parsimonious interpretation of the reading deficit in terms of a single deficit in word decoding. The possible source of this deficit may call for visual or phonological mechanisms, including Goswami's temporal sampling framework.

## Introduction

Reading aloud is a complex task that requires the synchronization of various subtasks or subcomponents which impinge on different ongoing fluxes of information. Thus, for reading to be effective orthographic stimuli have to be visually scanned, individual words have to be decoded and their corresponding entries in the phonological output lexicon have to be activated, a stream of utterances has to be produced and synchronized with the ongoing scanning of visual text and word meaning has to be decoded and maintained in short-term memory to place words in a context and reconstruct the sense of a sentence (or paragraph). By its very nature, scientific research tries to isolate different sub-tasks (using appropriate parameters), because complex behaviors are more difficult to study. This is also true in the study of reading and its disorders. For example, it is widely accepted that word level captures the difficulty of dyslexic children (Coltheart et al., 2001). Therefore, much research has aimed to examine single-word reading performance, often analysing the effects of psycholinguistic variables (such as word frequency or age of acquisition) by means of vocal reaction times (e.g., Ziegler et al., 2003) or lexical decision times (e.g., Martens and de Jong, 2006). As to the visual scanning of texts, research on eye movements has mainly examined the silent reading of lists of words or words embedded in simple sentences, and has avoided the complexities linked to the synchronization of voice outflow and limited the requests for text comprehension (Rayner, 2009).

The complexity of reading, with its multiple interacting subcomponents, represents an intriguing and challenging problem. In a recent study based on reading time measures (Zoccolotti et al., 2012), we contrasted the reading aloud of words presented singly or arranged in multiple arrays in control and dyslexic readers. Skilled readers showed a clear advantage with multiple over single items, indicating that they were able to process the subsequent visual stimulus while uttering the current target. By contrast, dyslexic readers did not show such an advantage and were actually slower in reading multiple than single items. We proposed that difficulty with arrays of words indicates a selective difficulty in integrating the multiple subcomponents of the reading task over and above the basic nuclear deficit in decoding words (Zoccolotti et al., 2012). Other studies (based on the Rapid Automatized Naming paradigm or RAN) also emphasized the particular difficulty of dyslexic children with the sequential nature of the naming task as opposed to the more artificial situation in which single items are presented one at a time and responded to (e.g., Georgiou et al., 2013; for a developmental analysis of this effect see also Protopapas et al., 2013). These observations raise interest in examining dyslexic readers' overall reading behavior, that is, from visual scanning to sentence utterance. Indeed, this might clarify two important aspects of developmental reading deficiencies.

First, the extent to which dyslexic readers are differentially impaired/spared in the different subcomponents that contribute to reading aloud could be evaluated. It could also be determined whether the requirement to integrate these multiple subcomponents causes, or at least contributes to, the reading difficulty. Some evidence concerning the second question has been cited above.

As to the first question, a large literature indicates that dyslexic readers have slower reaction times (RT) to visually presented words (and non-words) than control readers. This finding indicates that they have a basic deficit in decoding visually presented orthographic materials (e.g., Ziegler et al., 2003; Spinelli et al., 2005). By contrast, pronunciation time for words is much less affected (although it is not entirely normal), indicating a much weaker deficit in the production component (Davies et al., 2013; Martelli et al., 2013). Analysis of visual scanning generally indicates that dyslexic children show normal eye movement patterns with non-orthographic visual stimuli and have specific difficulty with orthographic materials in both irregular (e.g., Olson et al., 1983) and regular (e.g., Italian, the language studied here: De Luca et al., 1999) orthographies. There is growing evidence of difficulty also in the acoustic modality (i.e., dyslexia has been found to be associated with reduced perceptual sensitivity to amplitude envelope onset of sounds (e.g., Goswami et al., 2011; Leong et al., 2011). Within the temporal sampling framework (Goswami, 2011), the deficit in rise time sensitivity is associated with impaired low-frequency oscillatory mechanisms involved in parsing and perceiving syllables. Goswami (2011) noted that impairments in auditory entrainment are likely to have consequences for attention and also auditory–visual integration (are likely to affect attention and auditory-visual integration). Indeed, attentional deficits were also reported in dyslexic children (e.g., Hari and Renvall, 2001; Facoetti et al., 2010) and might contribute to the abnormal eye movement pattern shown in reading (see also Hawelka et al., 2010 for a link between word reading and eye movement control).

Even from this sketchy presentation, it is apparent that not all reading sub-components are equally impaired in dyslexic readers and that procedures which allow differentiating the level of involvement within the same general paradigm would be useful to fully describe the profile of reading slowness in these children.

In the present study, we moved in this direction by jointly examining the flow of eye movements and speech production in children with and without developmental dyslexia while they read aloud a short meaningful text. Interest in the inter-play between eye and voice during reading aloud was particularly keen in the first part of the last century (Buswell, 1920, 1921; Fairbanks, 1937; Tiffin and Fairbanks, 1937). These studies were aimed at separating peripheral and central phases of the reading process. By analysing reading errors in good and poor adult readers in relationship to eye movements, Fairbanks (1937) reported evidence that “*faulty eye movements cannot have caused the errors*” and concluded that “*the central processes in reading determine the nature of eye movements*” (Fairbanks, 1937). This position still has a large consensus today (see Rayner, 1998, 2009 for reviews).

Important observations in these studies concern the relationship between visual scanning and voice production during reading. Buswell (1921) effectively synthesized the characteristics of this phenomenon: *“A mature reader tends to maintain a comparatively wide average span between the eye and the voice, which at times may amount to the space occupied by seven or eight words. An immature reader, however, tends to keep the eye and voice very close together, in many cases not moving the eye from a word until the voice has pronounced it. Reading of this type becomes little more than a series of spoken words because there is no opportunity to anticipate the meaning in large units. An eye-voice span of considerable width is necessary in order that the reader may have an intelligent grasp of the material read, and that he may read it with good expression.”*

Fairbanks (1937) study of the eye-voice relationship was particularly thorough (although it has been rarely cited). He proposed that eye-voice lead (as compared to eye-voice span) was “*more descriptive of the eye-voice relation as it usually obtains*”; therefore, we will follow Fairbanks's terminology in the present work. Fairbanks (1937) examined the eye-voice lead in relationship to both spatial and linguistic parameters as well as the difficulty of words, defined by word frequency (“scarcity” in Fairbanks's terms) and the effect of stimulus length. The influence of these factors was clear in the pattern of eye movements as well as misreadings. Thus, “*in poor reading the influence of word scarcity is even greater*” than in superior readers, while “*word length has little effect upon good readers, but, to a degree at least, appears to be a measure of difficulty in inferior reading*.” Examining mispronunciations in a dynamic context, Fairbanks (1937) gave particular emphasis to multiple utterances, such as hesitations or repetitions with or without self-corrections. He noted that, when good readers repeated a word they did so after a misreading and made a self-correction. By contrast, poor readers often repeated correct words and nearly always failed to correct their errors.

Fairbanks (1937) observed that number of hesitations is also particularly important in discriminating good from poor readers even though they are difficult to measure as “*their determination is essentially qualitative*” and requires “*careful re-checking*.” Examining hesitations in association with eye movements, Fairbanks (1937) noted that “*one of the reasons for hesitation in poor reading is the necessity of regressing to words which give difficulty*” and “*… even though the determination of hesitations is a qualitative step, … diagnosis of reading ability by means of describing oral reading should include this measure*.”

These observations are particularly revealing because, since the classical work of Marshall and Newcombe (1966, 1973), analysis of misreadings in dyslexia has focused on the cognitive interpretation of errors characterized by the production of a single, isolated response, while multiple utterances “*such as circumlocutions, self-corrections and multiple responses*” did not interest researchers in part because they “*do not occur with great frequency*” (Coltheart, 1980) and in part because they do not easily fit in the cognitive category framework. However, hesitations and repetitions are time-consuming and may be responsible for a large portion of the characteristic slowing reported for dyslexic readers in regular orthographies (Italian: Zoccolotti et al., 1999; German: Wimmer, 1993). Indeed, it has been observed that children learning regular orthographies rarely make “classical” substitution errors and more often produce a slow and fragmented approach to the target word (Bakker, 1992). Hendriks and Kolk (1997) referred to this as “*sounding out behaviour*” to mark reliance on phonologically recoding the target; this is referred to as a reading behavior (rather than error) because, by the end of the process, the child is often able to utter the target correctly. Recently, we confirmed that many of the mispronunciations of Italian children with dyslexia are due to sounding-out behavior, which discriminates them from skilled readers (Trenta et al., 2013). Although Fairbanks is not explicit in his description of hesitations, they seem to resemble the time-consuming errors described by Bakker (1992) or the sounding-out behavior posited by Hendriks and Kolk (1997).

Going back to the eye-voice-lead question, Fairbanks emphasized the role of linguistic factors (such as word scarcity) vs. spatial factors (such as stimulus position). Furthermore, *he* noted that *“since one of the most obvious effects of difficulty in poor oral reading is hesitation of the voice, it appears that this is the cause of increase in eye-voice lead”*, and concluded that “*the amount of eye-voice lead is expressive of the combination of important factors involved in reading. It is a measure of the rate of recognition and assimilation*.” In the contrast between single and multiple word processing referred to above, we propose that a comparatively wide eye-voice lead should be taken as indication of the ability to integrate several sub-components of reading.

We have given particular space to the work of Fairbanks (1937) because it represents a very special case of a study in which an explicit attempt was made to cross-analyse the interplay between several sub-components of reading in both good and poor readers. After this pioneering period, interest in the vocal component of reading was seldom shown by eye movement research (for an exception see Morton, 1964), which relied almost completely on silent reading measures. Indeed, interest moved to perceptual or linguistic manipulations of written texts to determine the laws of eye movement control during reading, perceptual span and other topics related to cognitive processing during reading (for a review, see Rayner, 2009).

Consequently, research on eye-voice lead (or span) became rare. Some authors measured eye movements during oral reading by focusing on lexical processes and reading comprehension (Levin, 1979; Levy-Schoen, 1981). Inhoff et al. (2011) investigated the eye-voice span with the aim of refining models of eye movement control involved in reading aloud. Very recently, Pan et al. (2013) extended the study of the eye-voice lead to the multiple naming conditions typical of the RAN paradigm.

In the present study, we extended the early observations of Buswell (1921) and Fairbanks (1937) in measuring the eye-voice lead in Italian dyslexic readers and age-matched controls while they read aloud a meaningful text. By measuring eye movements and voice parameters together, we aimed to decompose the various reading sub-components to investigate which ones are more or less compromised in dyslexic readers. We also aimed to evaluate whether the integration of these multiple subcomponents contributes to generating the slowed reading that is found in children learning to read in regular orthographies (e.g., Wimmer, 1993; Zoccolotti et al., 1999). As a control condition, we also recorded eye movements during the silent reading of a text to evaluate the time advantage of silent over aloud reading.

As to eye movements, based on previous research in Italian children (De Luca et al., 1999, 2002) we expected to find problems associated with the number and duration of fixations. The distribution of fixation durations might be interesting in view of the temporal sampling framework (Goswami, 2011). Transposing Goswami's theory to the visual domain, we explored whether impaired low-frequency oscillatory mechanisms, which are important for mediating syllable perception, also have an effect at the visual level that is, on fixations in the same frequency range, with time durations of approximately 200 ms.

As to the vocal components, previous research indicates that articulation times are minimally affected in single word presentation in children with dyslexia (Davies et al., 2013; Martelli et al., 2013); however, no information is yet available on the articulation times of words immersed in a meaningful text, which was one of the aims of the present study. Finally, we also examined reading accuracy. In view of Fairbanks's (1937) observations, we focussed on sounding out behavior and pauses (which are time-consuming behaviors) in addition to classical substitution errors.

## Materials and methods

### Participants

Participants included 16 dyslexic readers and 16 chronological age-matched control readers. The school where participants were enrolled collected written informed consent provided by each student's family, as part of an agreement between the school and the Sapienza University of Rome. Groups were comparable for age, gender, and non-verbal IQ level (see Table 1). Each of the dyslexic readers scored at least 1.65 *SD* below the norm for either speed or accuracy on a standardized reading test (MT Reading test, Cornoldi and Colpo, 1995). In this test, the child reads a text passage aloud with a 4-min time limit; reading time (s/syllable) and accuracy (number of errors, adjusted for the amount of text read) are scored (see Table 1 for both raw and normalized values). Considering the reading speed raw data, the average reading time of the two groups was 0.22 (typically developing readers) and 0.43 s/syllable (children with dyslexia). This indicates slowing with a factor of 1.95 (below see further comments on this ratio). Non-verbal IQ level was assessed using Raven's Colored Progressive Matrices. All children scored well within the normal limits according to Italian norms (Pruneti et al., 1996). All participants had normal or corrected-to-normal visual acuity.

**Table 1:** **Summary statistics for the two groups of participants: mean age (in years, with range in square brackets); number of female and male participants; mean *z*-scores (and *SD* in parentheses) on Raven's Colored Matrices; mean reading times and mean number of errors on the MT Reading test; mean *z*-scores for reading time and accuracy on the same test**.

	**Dyslexic readers**		**Control readers**		***p*-level**
Age	11.9	[11.3−12.9]	11.6	[11.1−13.4]	n.s.
Males/females	9/7		10/6		n.s.
Raven test	−0.7	(0.6)	−0.3	(1.0)	n.s.
Reading time (s/syllable)	0.43	(0.14)	0.22	(0.02)	<0.001
Reading accuracy (number of errors)	22.5	(7.9)	4.7	(2.6)	<0.001
Reading time (*Z* score)	−2.3	(1.6)	0.2	(0.3)	<0.001
Reading accuracy (*Z* score)	−3.4	(1.8)	0.2	(0.5)	<0.001

Probability values for t-tests (except for gender, for which the Chi square test was used) comparisons between the two groups are reported (p < 0.01 values are considered significant after Bonferroni correction).

### Apparatus and procedure

Eye movements from the dominant eye were recorded in binocular vision via an SR Research Ltd. Eye Link 1000 eye tracker (SR Research Ltd., Mississauga, Ontario, Canada) sampling at 1000 Hz, with spatial resolution of less than 0.04°. Head movements were avoided by using a headrest, but the chin was left free. The text was displayed on a 17″ CRT monitor at a viewing distance of approximately 57 cm. Screen resolution was 1024 × 768; refresh rate was 85 Hz. The ambient illumination level was kept constant across recordings by artificial lighting. A nine-point calibration procedure was run before the passage was shown. The calibration targets were presented randomly in different positions on the screen. Appearance of the text on the screen was triggered by fixation of a cross in the upper left position corresponding to the blank space adjacent to the initial letter of the passage.

Voice was digitally recorded by a system mounting a Shure microphone, a pre-amplifier, an E-MU sound card, and an ASIO driver, which was interfaced to the eye tracker by Eye Link Experiment Builder software.

Separately for each participant, eye movements and voice utterances were simultaneously recorded and stored in the same PC directory; the output consisted of two parallel recordings: an eye movement dataset and a sound file in wav format. Eye movements and audio recordings were synchronized by the eye-tracking device after eye-movement calibration. The voice-line recording onset and offset was automatically overlaid on the timeline of the eye movements recording output.

### Materials

Two text passages were used, one for reading aloud and one for silent reading; both were adapted from Aesop's fables and were appropriate for the age range of the observers. Each text subtended a visual angle of 22 × 13°, displayed at the center of the screen horizontally and at 5° from the top edge of the screen vertically. Passages were written in Times New Roman font (because it is similar to functional reading texts), with black letters on a white background. Average center-to-center letter distance subtended 0.4°. The two texts had the same number of lines (14) and were also matched for number of syllables and characters; finally, the content words of the two passages had comparable mean word frequency. The first line of both texts contained a filler sentence (ending with a full stop), which was not used in the analyses.

Only the three-line sentence, from the second to the fourth line of the text, for reading aloud was used for in-depth analyses of eye and voice parameters and will be described in detail. It contained 31 words (on average 10.3 words per line) and 173 letters (inter-word spaces included). Average word length was 4.6 letters. The mean log frequency of the words was 3.02 (range 0.7–4.9), according to a corpus of the Italian written language of 3,798,275 occurrences (CoLFIS; Bertinetto et al., 2005).

The order of the reading condition was randomized across participants. A cross was displayed in the upper-left quadrant of the screen (2° to the left of the first letter of the first text line) and served as the initial fixation target; the offset of the cross and the simultaneous onset of the display containing the passage was automatically triggered by the eye-tracking device when the participant steadily fixated the cross for at least 150 ms. Participants were asked to read each passage at their normal rate; in the reading aloud condition, the passage remained on the screen until the end of the last word uttered. To reliably assess the last fixation of silent reading, participants had to look at a two-figure number immediately after they finished reading, name the number aloud, and communicate that they had finished reading. The number subtended 0.25° and was displayed in the bottom right corner of the screen. To evaluate general comprehension, at the end of the reading participants were asked to answer questions; there was a yes/no question and an open question for each of the two text passages.

### Data analysis

#### Eye movements

Eye movement data were processed using the EyeLink Data Viewer software (SR Research Ltd., Mississauga, Ontario, Canada). Total viewing time (i.e., time needed to visually examine the text, from the beginning of the first fixation until the end of the last fixation) was computed for the whole passage (13 lines) for both oral and silent reading conditions. Only the above-mentioned three-line sentence of the reading aloud text was analyzed in detail: Besides total viewing time, total fixation time (i.e., the sum of all fixation durations), total number of fixations, mean fixation duration, forward saccade mean amplitude (in degrees of visual angle) and percentage of regressions were measured; fixation positions were mapped over the screenshot of the three-line sentence to determine which graphemes in the passage were fixated.

#### Audio tracks

Audio recordings were processed off-line using Audacity 2.0.2 software. The temporal onsets and offsets of utterances and the silent pauses were marked along the timeline interface of each audio file for the three-line sentence using a mixed criterion of visual inspection of the waveform image and listening to the audio track (the minimum duration for a pause to be reliably detected combining visual and audio information was 40 ms). In fluent readings, between-word pauses were rare; the final and initial phonemes of consecutive word utterances merged due to co-articulation.

Correct utterances, misreadings, and pauses between and within words were identified and labeled along the file timeline.

Total reading aloud time, total pronunciation time (i.e., the sum of utterances excluding pauses and including all kinds of misreadings), total duration of pauses (the sum of the durations of all silent pauses), mean duration of single silent pauses, number of silent pauses, and mean utterance duration for correctly read content words were scored (errors as well as sounding-out were excluded) for the three line sentence. Moreover, total reading aloud time was also computed for the whole text.

Accuracy was scored by considering the following categories of errors: sounding-out behavior (i.e., progressive approximation toward the correct utterance of the whole word made through sounding-out parts of the word; e.g., [‘ta ‘tavolo] instead of [‘tavolo], “table”), word substitutions, word omissions or insertions and non-word production.

#### Eye-voice lead

The eye-voice lead was measured by detecting the within-word uttered phoneme for each fixation point in the first three lines of text. The lead was measured as the spatial distance, namely, number of letters, between the fixated grapheme and the simultaneously uttered phoneme. The single letter (grapheme) was chosen as the spatial unit (inter-word spaces counted as one letter). Each letter (and inter-word space) of the three-line sentence was progressively numbered from 1 to 173. Consistently, phoneme positions were progressively numbered along with the printed letters. Because of the transparency of the Italian writing system, there was a 1:1 correspondence between the number of graphemes and phonemes for 27 out of 31 words; three of the remaining four items had a number of phonemes equal to N–1 with respect to the corresponding printed word, and one had N–2 phonemes.

**Figure 1:**
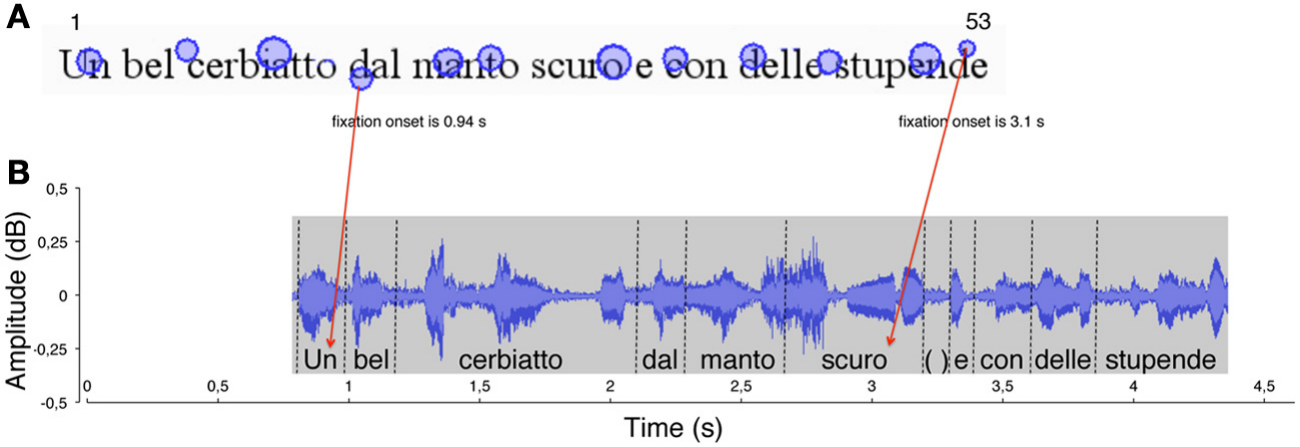
**An example of the method used to measure the eye-voice lead is reported for a typically developing reader. (A)** In the spatial overlay of the eye movement pattern, the circles represent the localizations of eye fixations over the first line of the sentence. The size of the circles is proportional to fixation duration. The numbers 1 and 53 represent (for example) the first and last labels for letter position number in the line. The onset time of two fixations is indicated. **(B)** The waveform of reading aloud is shown as a function of time. The vertical dotted segments delimit the single word utterances, labeled along the timeline. Red arrows indicate the temporal correspondence between a fixation (the grapheme “d,” of the word “dal,” letter number 18) and the phoneme that was uttered at the time of fixation (the phoneme/u/, letter number 1). Each fixation (e.g., “d,” of the word “stupende,” number 52) was linked to the phoneme uttered at the moment of fixation onset (the phoneme/o/ of the word “scuro,” number 32). The eye-voice lead was obtained by subtracting the phoneme position number from the grapheme position number for all of the grapheme-phoneme pairs (in the latter example: 52–32 = 20 letters).

Then, each gaze position (i.e., fixation on a grapheme) was matched with the “voice position,” that is, the phoneme uttered at the temporal onset of fixation. This was carried out by identifying the graphemes that were fixated on the eye movement output (see Figure 1A) and the phonemes that were simultaneously uttered (at each fixation onset) in the sound file output (see Figure 1B). For each of the fixation points, the corresponding grapheme position (number) was computed. Then, the phoneme uttered simultaneously with fixation onset (as determined by the time line of the recording) was “anchored” to the fixated grapheme (see red arrows in Figure 1) so that pairs of *grapheme position number* and *phoneme position number* were obtained for all fixations made during reading (N fixations = N pairs).

The eye-voice lead was measured at each fixation point as the difference between phoneme and grapheme positions in space (namely, difference between simultaneous phoneme and grapheme numbers). The individual eye-voice lead (across the three-line sentence) was measured in two ways: first, by averaging the difference values across the grapheme-phoneme pairs; second, by subtraction of the intercept values of the voice from the eye regression lines.

#### Statistical comparisons

Comparing groups with generally different performances by standard parametric analyses is problematic because groups may show systematic differences in variability (thus, violating the assumption of homogeneity of variance). However, it is generally believed that ANOVAs or Student *t*s are sufficiently robust comparisons for moderate violations of homogeneity. Therefore, *t*-tests for independent samples were run for all group comparisons and the Bonferroni correction was adopted for multiple comparisons.

To evaluate which variables contributed (and how much) to the impaired performance of the dyslexic children, standardized (i.e., Cohen's *d*) and unstandardized (i.e., ratio) effect sizes were computed. The first ones convey the size of an effect relative to the variability in the samples. Reference points for small, medium, and large effects are considered 0.20, 0.50, and 0.80, respectively. However, also unstandardized measures, such as ratios, are of particular interest because they allow comparing performances in proportional terms across conditions (and groups) showing very different variability. To this aim, the mean values of the dyslexic children were divided by the values of the control readers to obtain the ratios (except in the case of forward saccades amplitude, where the inverted ratio was calculated). In all cases, ratios greater than 1 indicate that dyslexic readers performed worse.

Pearson correlations were run between eye-voice lead data and MT Reading Test, eye, and voice parameters for the whole group of children.

## Results

### Reading performance: voice data

The data based on the audio tracks are presented in Table 2. Total reading aloud time was significantly longer for dyslexic than control readers for both the three-line sentence and the total text (with similar ratios of about 2). Total pronunciation time (i.e., time excluding pauses) also distinguished the two groups, but with a lower ratio (1.4). For all these measures, *d* values greatly exceeded the reference for a large effect (0.80).

**Table 2:** **Group results for reading aloud data based on the audio tracks**.

	**Dyslexic readers**		**Control readers**				
	**Mean**	***SD***	**Mean**	***SD***	***t***	**ratio**	***d***
Total reading aloud time	113.6	31.8	60.2	5.6	6.46	1.9	2.3
Total reading aloud time	22.1	7.4	11.4	1.5	5.67	1.9	2.0
Total pronunciation time	14.6	2.3	10.3	0.9	6.82	1.4	2.5
Mean single word utterance duration	0.608	0.054	0.489	0.046	6.76	1.2	2.4
Total duration of silent pauses	7.5	6.3	1.1	0.8	4.07	7.1	1.4
Mean duration of silent pauses	0.539	0.208	0.363	0.131	2.88^*^	1.5	1.0
Number of silent pauses	13.5	5.9	3.0	1.9	6.74	4.5	2.4

The first row refers to the full passage (13 lines); the remaining rows to the three-line sentence. Time measures are reported in seconds. Total reading aloud time goes from the beginning of the first utterance to the last pronunciation (pauses and misreadings included). Total pronunciation time is the sum of the duration of all utterances (pauses excluded). The mean pronunciation time was computed for correctly uttered content words. Total duration of silent pauses is the sum of the durations of periods in which no utterance was made. Means and SDs are reported. T and probability values for statistical comparisons between groups (p < 0.006 is considered significant after Bonferroni correction) and ratios between the mean values of the two groups are also reported. All p's < 0.001, except

* *= 0.007*.

Utterance duration of single words (for content words correctly pronounced in the three-line sentence) was 119 ms slower in dyslexic with respect to control readers; notably, this effect was highly significant even though it indicated only a quantitatively modest 20% slowing (ratio = 1.2). When we restricted the computation to those words that were correctly pronounced by all children in the two groups, the difference was still present; thus, dyslexic readers needed 655 ms (*SD* = 66 ms) and control readers, 575 ms (*SD* = 83 ms); the 80 ms difference was highly significant (*t* = 3.02, *p* < 0.01) but again indicated a rather small effect (ratio = 1.1). Note that although the ratio was low, the standardized effect size for this measure was considerably higher and above the cut-off of 0.80 for a large effect size (see Table 2).

**Figure 2:**
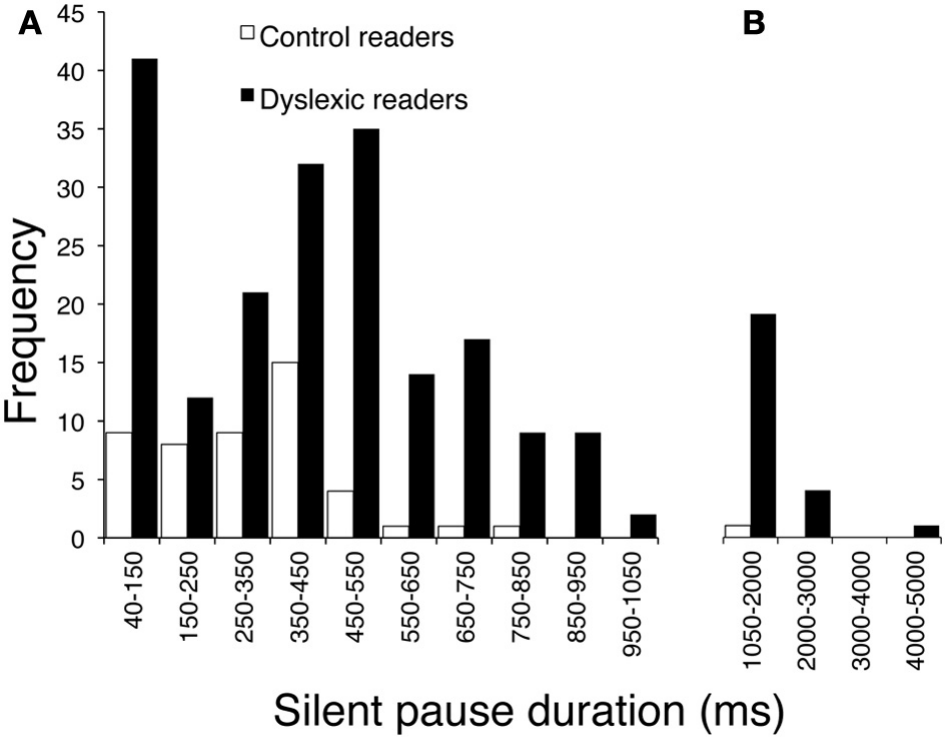
**Distribution of frequency for pause durations, separately for children with dyslexia and typically developing readers. (A)** Intervals on the abscissa by 100 ms each (except for the first one, which ranges from 40 to 150 ms). **(B)** Intervals by 1000 ms each.

The children with dyslexia made many more pauses (ratio 4.5) and spent much more time in silence (with a 7.1 ratio for total duration of pauses) than control readers. Mean pause duration was slightly longer in dyslexic readers (this comparison just failed to be significant after Bonferroni correction). The frequency by duration of pauses distribution in the two groups is presented in Figure 2. The mode of the distribution was the 350–450 ms interval for control readers; the distribution was more complex for dyslexic readers, with two peaks at 40–150 ms and 450–550 ms (Figure 2A) and a third peak at very long intervals (Figure 2B; note the different interval scale).

#### Comments

When overall reading aloud time was considered, there was a remarkable group difference (90% or 1.9 ratio) in the time taken to read the three-line sentence. Notably, the same ratio was obtained in the MT Test reading time measure (1.9; based on data from Table 1). A very similar figure was reported by Martelli and co-workers who averaged six different studies (Martelli et al., 2013). Overall, a factor of 2.0 can be considered as a reference point to interpret reading time data as well as other reading parameters.

A quantitatively small (20% or 1.2 ratio), but highly significant, difference was present for pronunciation time of correctly read single words. Notably, all misreadings were excluded from these computations; therefore, the small lengthening in voice utterance must be taken as genuine. Furthermore, the difference remained when the computation was restricted to the words that were correctly articulated by all children, indicating that the effect on articulation times did not depend on differences in the actual words uttered. Interestingly, individual variability in utterance duration was very low and not different in the two groups of children. This is partially in keeping with the idea that execution times yield smaller individual differences than cognitive times; furthermore, execution times are assumed as a constant in models of decision processing (e.g., *difference engine model* by Myerson et al., 2003). Notably, a considerable difference was detected between unstandardized (ratio) and standardized effect sizes. Thus, the ratio indicated a very small group difference, whereas the standardized measure of effect size (due to the very low variability) indicated a very large group effect for this parameter (*d* = 2.4).

Finally, dyslexic children spent much more time in silent pauses than control readers. This effect was due to a large difference in the number of pauses and a smaller difference in terms of pause length. However, as shown in Figure 2, very long pauses were present only in the dyslexic readers. When total pronunciation time without pauses was considered, the ratio between the two groups was considerably smaller (1.40). Thus, frequent and long pauses represent an important characteristic of reading in dyslexia.

### Reading performance: accuracy and comprehension

Table 3 reports the percentages of misreadings for the two groups of children. More than half of the inaccurate utterances in both groups indicated sounding-out behaviors. Notably, dyslexic children engaged in this behavior nearly in 10% of cases. They also made few word substitutions (4.4%) and rare omissions or insertions of words (0.8%); in a few instances, their productions resulted in non-words (0.8%). Controls engaged in a much smaller proportion of sounding-out behaviors and made fewer word substitutions and never omitted or inserted a word; furthermore, they never produced a non-word. Effect sizes (whether unstandardized or standardized) were all very large, particularly in the case of sounding-out behavior (i.e., dyslexic readers engaged in this behavior nearly six times more than control readers).

**Table 3:** **Group results for misreadings**.

	**Dyslexic readers**		**Control readers**				
	**Mean**	***SD***	**Mean**	***SD***	***t***	**ratio**	***d***
Sounding-out behaviors (%)	9.5	4.8	1.6	2.0	5.90	5.9	2.15
Word substitutions (%)	4.4	3.7	1.2	2.3	2.95	3.7	1.04
Word omission or insertion (%)	0.8	1.4	0.0	0.0	–	–	–
Non-word production (%)	0.8	1.4	0.0	0.0	–	–	–
Total errors (%)	15.5	7.7	2.8	3.3	6.09	5.5	2.14

Both the percentage of total errors (last row) and the percentages for each category of misreadings (sounding-out behavior, word substitution, word omission or insertion, and non-word production) are presented. The percentages are calculated with respect to the total number of words in the three-line sentence. Means and SDs are reported. T-test comparisons between groups (p < 0.017 is considered significant after Bonferroni correction) and the ratios between values of the two groups are also reported. All p's at least < 0.01.

At the end of the reading task, both groups responded correctly to the open and closed questions: 15 out of 16 typically developing children responded well to the open question and 13 to the closed question. The figures were 14 (out of 16) and 15 in the case of dyslexic children. In silent reading, results were quite similar: 14 control readers responded well to the open question and 10 to the closed question. The figures were 14 (out of 16) and 12 in the case of dyslexic readers.

#### Comments

Misreadings mostly indicated a halting, but effective (i.e., eventually leading to correct pronunciation), approach to the target words (sounding-out behavior). This occurred much more often in dyslexic than control readers (i.e., almost six times more often). These data confirm the predominance of time-consuming errors recently reported in Italian dyslexic children (Trenta et al., 2013). Surprisingly, this ratio is quite similar to the one originally observed by Fairbanks (1937) in English-speaking children; in that study, poor readers made 5.6 more hesitations than good readers (i.e., 8.87 vs. 1.56%).

Word substitution errors also discriminated between the two groups but were relatively few in absolute terms, confirming previous observations in regular orthographies (Wimmer, 1993; Zoccolotti et al., 1999).

Finally, in agreement with previous data collected in silent reading conditions (De Luca et al., 1999), the slow and inaccurate reading of dyslexic children did not prevent adequate text comprehension.

### Eye movements during reading

**Figure 3:**
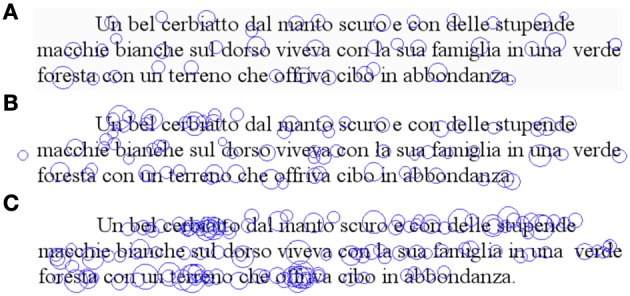
**Spatial overlay of the eye movement pattern during reading of the three-line sentence**. Fixation points (circles) are indicated for a typically developing reader **(A)**, for a child with mild dyslexia **(B)** and a child with severe dyslexia **(C)**.

Representative eye movement patterns of children with mild and severe dyslexia are presented in Figures 3B,C, respectively; the high number of fixations is evident with respect to performance of a typically developing reader (Figure 3A).

Table 4 reports eye movement results for dyslexic and control readers.

**Table 4:** **Group results for eye movement data**.

	**Dyslexic readers**		**Control readers**				
	**Mean**	***SD***	**Mean**	***SD***	***t***	**ratio**	***d***
Total viewing time—aloud	114.1	32.0	60.5	5.5	6.60	1.9	2.3
Total viewing time—silent	91.8	25.1	50.0	7.8	6.34	1.8	2.2
Total viewing time	22.5	7.8	11.5	1.4	5.52	2.0	2.0
Total fixation time	20.7	7.3	10.4	1.2	5.52	2.0	2.0
Number of fixations	72.2	20.9	39.9	5.7	5.95	1.8	2.1
Mean fixation duration	0.29	0.04	0.263	0.026	1.86^*^	1.1	0.6
Forward saccades mean amplitude	1.68	0.30	2.02	0.24	3.50	1.2	1.2
Percentage of regressions	27.7	5.8	15.1	8.7	4.82	1.8	1.7

The top part of the table refers to the full passage (13 lines) separately for aloud and silent reading, the bottom to the three-line sentence for reading aloud. Time is reported in seconds, saccade amplitude in degrees. Total viewing time indicates the time starting from the first fixation and ending with the last fixation. Total fixation time was the sum of all discrete fixation durations. Number of fixations includes all fixations (i.e., both first and second pass fixations). Mean fixation duration is the average of all discrete fixations that lasted more than 100 ms. Saccade amplitude is averaged across all rightward saccades, except for small corrective saccades occurring during the return sweeps. Means and SDs are reported. As for between- group comparisons, t-values are reported (p < 0.007 is considered significant after Bonferroni correction). The last column reports the ratio between the means of the two groups. The values of children with dyslexia were divided for the values of typically developing readers to obtain the ratios, except in the case of forward saccades amplitude, where the inverted ratio was calculated. In all cases, ratios greater than 1 indicate worse performance of children with dyslexia. All p's < 0.001, except

* *(n.s.)*.

The first part of the table reports total viewing time for the whole passage read aloud and that read silently. Both groups of children spent less time scanning the text when reading silently than when reading aloud (dyslexic readers: *t* = 2.20, *p* < 0.01; control readers: *t* = 4.41, *p* < 0.001). In absolute values, the difference was greater in dyslexic children (22.3 s) than in control readers (10.5 s); however, the ratio between aloud and silent reading was quite similar in the two groups (dyslexic children = 1.24; control readers = 1.21). Also, the ratio between the two groups for viewing time was similar for reading aloud (1.9) and silent reading (1.8). Standardized effects were very large (over 2) and similar for aloud and silent reading.

**Figure 4:**
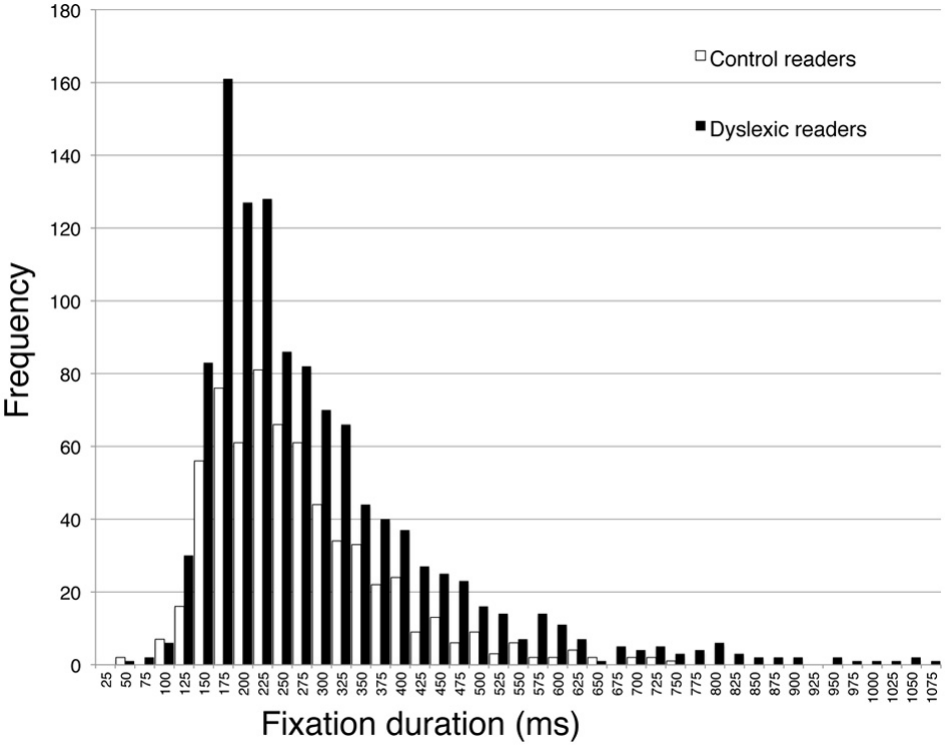
**Distribution of frequency for fixation duration, separately for dyslexic and control readers**. Intervals on the abscissa by 25 ms each.

The second part of Table 4 reports eye movement results measured in greater depth for reading aloud the three-line sentence. Total viewing time data are consistent with those of the whole passage. Dyslexic readers showed a significantly higher number of fixations and a higher percentage of regressions and smaller forward saccade amplitude with respect to control readers. No significant difference was detected for fixation duration. The distribution of fixation durations for the two groups of children is presented in Figure 4: note that the two groups show a very similar number of short fixations (<100 ms), while the number of longer fixations is higher for dyslexic children and highest in the 175–225 ms time interval.

Ratios between the two groups were close to a factor of 2.0 for number of fixations and percentage of regressions and were lower for fixation duration and saccade amplitude. The *d* values were all above the critical value for a large effect, apart from mean fixation duration.

#### Comments

Both groups of children had longer viewing times when reading aloud than when reading silently. The group differences were quantitatively more marked in the former case but were proportionally constant across the two conditions, i.e., the children with dyslexia were slower than typically developing readers by about a factor of 2 for reading aloud as well as for silent reading. In general, these data confirm well-known observations (e.g., Anderson and Swanson, 1937), but the authors focussed on absolute rather than proportional effects. This indicates a basic problem in analysing reading data. Whenever manipulations of the task (such as, here, reading aloud vs. silent reading) produce an increase in the time necessary to perform the task, group differences also increase; thus, absolute differences in performance change while proportional differences remain stable. Possible interpretations of this pattern of findings will be examined in the Discussion (see below).

As to the pattern of eye movements, children with dyslexia showed a slow and fragmented reading pattern characterized by many fixations, smaller forward saccade amplitude and a higher percentage of regressions with respect to typically developing readers. By contrast, there was a non-significant difference in mean fixation duration between the two groups. Using 200 ms as a standard temporal fixation threshold (Salthouse and Ellis, 1980), Figure 4 shows that frequency of fixations around this temporal window was particularly high in dyslexic children. Ratios indicate that number of fixations and percentage of regressions were the parameters that distinguished most clearly between the two groups (with values close to the 2-ratio reference), whereas lower ratios emerged for fixation duration and saccade amplitude. Therefore, it seems that the main difference in eye movements between the two groups of children is related to number of movements rather than saccade amplitude or fixation duration.

### Eye-voice lead

**Figure 5:**
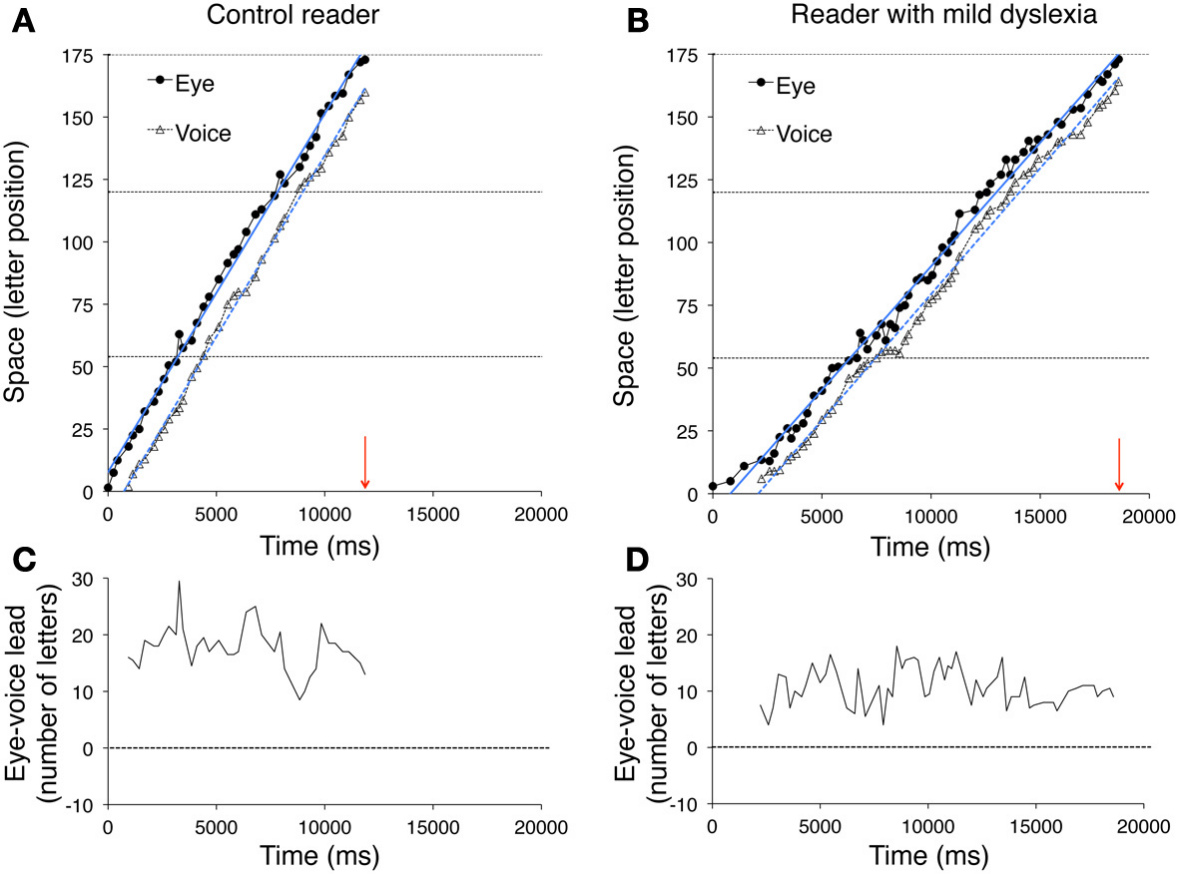
**The pairs of fixated grapheme/uttered phoneme positions are plotted as a function of time for a typically developing reader (A) and a child with mild dyslexia (B)**. The horizontal dashed lines represent the end of the first, second and third line of the sentence, from bottom to top, respectively. The arrows on the abscissa indicate the overall time necessary to read the three lines for each reader. Filled circles and open triangles represent fixations (i.e., eye position data) and utterances (i.e., voice position data), respectively. The ordinate reports the spatial position measured in number of letters (range: 1–173). The vertical space between simultaneous graphemes and phonemes expresses the eye-voice lead as a function of time. Eye and voice positions are fit by regression lines whose slopes indicate the reading rate, separately derived from eye movements and utterances. The intercepts on the ordinate axis (not visible in the figure because the negative part of the axes is not shown) may be used to compute the overall eye-voice lead. In plots **(C,D)**, the eye-voice lead is represented as a function of time as letter difference between pairs of simultaneous grapheme and phoneme positions.

**Figure 6:**
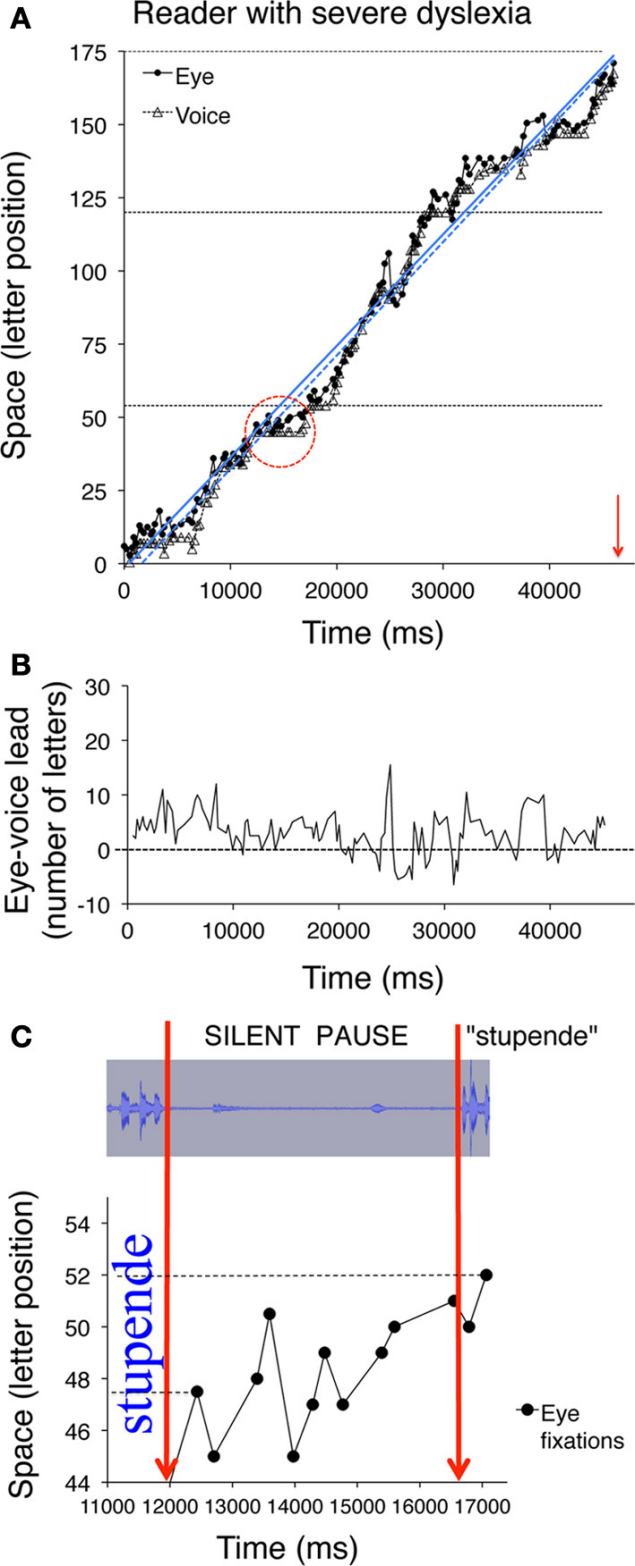
**Eye and voice data are presented for a child with severe dyslexia**. Similar to Figures 5A,B, the pairs of fixated grapheme/uttered phoneme positions are plotted as a function of time in panel **(A)**. Note that the scale on the abscissa of panel **(A)** is twice that of Figures 5A,B, therefore the slope indicates a much slower reading rate than that of the child with mild dyslexia represented in Figure 5B. In panel **(B)** (as in Figures 5C,D) the eye-voice lead is represented as a function of time as letter difference between pairs of simultaneous grapheme and phoneme positions. The **(C)** inset (obtained by zooming on the marked area of plot **A**) represents a long silent pause made by the child, during which eye fixation scanning is characterized by eight rightward saccades and three regressions in the word “stupende” (English translation: “wonderful”) before pronouncing it. The top part of the inset represents the spectral image of the audio track corresponding to the time interval represented in the bottom part, which corresponds to the pattern of fixations made on the word. Note that both the second and the fifth fixations (at letter position number 45) follow a regressive eye movement that brings the gaze to the inter-word blank space.

Figure 5 illustrates data for a control reader (left) and a child with mild dyslexia (right), respectively. A third example (Figure 6) reports data of a particularly severe case of dyslexia.

In the upper plots, the position numbers of the fixated grapheme/uttered phoneme pairs are plotted as a function of time for the three lines of text: filled circles refer to eye fixation data (referred to as “eye”) and open triangles to the corresponding utterance data (referred to as “voice”). The ordinate axis reports the spatial position as number of letters. Note that eye data are always above voice data, indicating the leading of the eye.

Two regression lines are drawn separately for the fixation and utterance data; they allow computing the eye-voice lead independent of local variability at specific points of the text. Reading rate is marked by the slopes of the regression lines for both eye and voice data. Slopes are higher for the skilled reader (Figure 5A) than the child with mild dyslexia (Figure 5B) and the severe case of dyslexia (Figure 6A).

In all upper graphs, voice pauses can be detected where consecutive voice data points (triangles) align parallel to the abscissa. This is rare in skilled readers but clearer in dyslexic readers; one very long pause made by the child with severe dyslexia is highlighted in inset of Figure 6C. Note the multiple fixations (most of which followed rightward saccades) that characterized the scanning of the word “stupende” (“wonderful”) by the child while he silently analyzed the text.

The eye-voice lead measured at each fixation point as the difference between phoneme and grapheme positions in space is presented as a function of time in the lower graphs of Figure 5 and the middle graph of Figure 6. Note that all values are positive, indicating that pronunciation of a word started after it had been visually inspected. Occasionally, however, in severe cases of dyslexia (including the one presented in Figure 6) there are also negative values. This occurred when the reader completed the utterance of a word while his gaze turned backward (second pass reading) to previously fixated portions of text.

Table 5 reports individual and group data for the slopes of eye movements and voice and the eye-voice lead. The eye slope was almost equal to the voice slope in all participants.

**Table 5:** **Eye-voice lead: mean individual data and group results**.

	**Reading rate (letters/s)**		**Eye-voice lead (number of letters)**		
	**Eye slope**	**Voice slope**	**Averaged eye-voice position difference**	***SD***	**Intercepts differences**
D1	10.0	10.0	10.7	3.4	11.2
D2	8.2	8.2	9.8	4.7	10.6
D3	9.1	9.1	9.6	4.6	10.2
D4	9.2	9.3	8.9	5.4	9.5
D5	10.3	9.9	11.2	4.7	9.0
D6	11.0	11.0	9.0	4.3	8.8
D7	8.4	8.4	8.0	4.2	8.6
D8	6.2	6.3	7.1	3.0	8.6
D9	10.4	10.4	8.1	5.1	8.3
D10	9.6	9.7	7.5	4.1	8.1
D11	11.6	11.7	6.9	3.6	7.8
D12	9.3	9.3	7.6	4.0	7.6
D13	6.3	6.5	7.1	4.2	9.1
D14	5.7	5.7	7.5	4.1	7.4
D15	6.7	6.7	4.9	2.8	5.1
D16	3.8	3.9	3.0	4.0	4.7
**Dyslexic readers mean**	**8.5**	**8.5**	**7.9**		**8.4**
***SD***	**2.2**	**2.1**	**2.1**		**1.7**
C1	17.9	18.4	16.5	4.8	19.3
C2	14.1	14.2	19.1	4.7	19.2
C3	14.4	14.5	17.7	4.0	18.4
C4	18.4	18.4	16.7	4.5	16.8
C5	18.0	17.9	16.9	4.2	16.4
C6	15.8	16.0	13.3	5.5	14.8
C7	18.2	18.1	15.2	5.5	14.4
C8	14.5	14.7	12.2	4.7	14.0
C9	16.5	16.2	15.4	4.5	13.8
C10	17.9	18.2	12.1	4.3	13.6
C11	15.4	15.5	11.3	5.5	12.3
C12	14.3	14.3	12.1	3.8	11.9
C13	13.1	12.8	13.0	3.5	11.6
C14	11.8	11.8	9.7	4.5	9.7
C15	12.6	12.5	9.7	4.1	9.3
C16	12.4	12.4	9.5	2.9	9.3
**Control readers mean**	**15.3**	**15.4**	**13.8**		**14.0**
***SD***	**2.3**	**2.3**	**3.1**		**3.3**
***t***	**8.68**	**8.65**	**6.36**		**6.02**
**Control/Dyslexic readers ratio**	**1.8**	**1.8**	**1.7**		**1.7**

*The first two columns report the reading rate (i.e., the number of letters read per second) as measured by eye and voice slopes, respectively. The mean eye-voice lead, reported in the third column, was obtained by averaging subtraction values of voice position data from eye position data (as in Figures 5C,D, 6B); in the fifth column the lead is reported as the difference between the intercepts of the eye and voice regression lines. T values for statistical comparisons (p < 0.012 is considered significant after Bonferroni correction) and ratios between the values of the two groups are also reported. All p's < 0.001*.

The mean eye-voice lead measured by averaging the differences between gaze and voice (as in Figures 5C,D, 6B) was significantly smaller in dyslexic (7.9 letters) than in control (13.8 letters) readers. Computing the eye-voice lead based on the difference between the intercepts of the regression lines produced very similar results: the eye-voice lead was significantly smaller in dyslexic children (8.4 letters) than in control readers (14.0 letters). Note that intra-individual variability (as assessed by the averaged eye-voice position) was similar in the two groups.

#### Comments

For all individuals in both groups, the values of the slope of the regression lines separately fitting the eye data and the voice data were almost equal (thus, the lines are parallel in all cases). This indicates that, regardless of reading skill, eyes and voice proceeded in synchrony, with a pacing which, despite local variability at specific points of the text (such as in the case of pauses) was kept relatively constant across lines of text. The voice lead showed a ratio between dyslexic and control readers (i.e., 1.7) in line with other key parameters, such as total reading time.

In good readers, the eye and voice regression lines were well spaced (about 14 letters or 5.6° of visual angle on the screen); by contrast, the distance was short in dyslexic children (about 8 letters or 3.2°). Thus, in dyslexic readers, the spatial distance between the uttered phoneme and the fixated grapheme was closer (about half in terms of number of letters or degrees) than in skilled readers. Notably in dyslexic readers the overall processing time of the same three lines (i.e., 22.1 s) was about twice that of control readers (11.4 s). Thus, the time by space product was almost constant in the two groups.

### Eye-voice lead: correlation with eye and voice data

Correlations between eye-voice lead and eye and voice parameters as well as performance on the MT reading test are reported in Table 6 for the whole group of children; after Bonferroni correction, correlations with *p* < 0.003 are considered significant. The eye-voice lead correlated highly with reading time and accuracy in the MT test. As for voice data, the eye-voice lead correlated highly with total reading aloud and total pronunciation time. Correlation with mean word utterance duration was also significant. Furthermore, eye-voice lead correlated highly with the number of pauses and their summed duration but only marginally with the mean duration of a single pause. As for eye data, correlation was high with viewing times, number of fixations, total fixation time and percentage of regressions, but was lower, and non-significant, with mean fixation duration and amplitude of rightward saccades.

**Table 6:** **Pearson correlations between the eye-voice lead (based on averaged eye-voice position differences) and various parameters of functional reading (MT Reading test), reading aloud, and eye movement data**.

		**Total sample**
MT Reading Test	Reading time	−0.76
	Accuracy	−0.77
Voice data	Total reading aloud time (whole passage)	−0.78
	Total reading aloud time	−0.77
	Total pronunciation time	−0.73
	Mean word utterance duration	−0.61
	Total duration of silent pauses	−0.70
	Mean duration of silent pauses	−0.50^*^
	Number of silent pauses	−0.83
Eye data	Total viewing time (whole passage)	−0.78
	Total viewing time	−0.76
	Total fixation time	−0.76
	Number of fixations	−0.76
	Mean fixation duration	−0.37^◦^
	Forward saccades mean amplitude	0.40^◦^
	Percentage of regressions	−0.69

The values of the r coefficient are marked for p < 0.003, considered significant after Bonferroni correction. Voice and eye data refer to the first three lines of text unless otherwise specified. All p's < 0.003, except for

* = 0.003 and

◦ (n.s.).

Moreover, correlations were calculated considering silent reading of the whole text. The total viewing time for aloud reading was highly correlated with that for silent reading (*r* = 0.85, *p* < 0.001). Finally, the eye-voice lead in reading aloud was highly correlated with the total viewing time in silent reading (*r* = −0.75, *p* < 0.001).

#### Comments

The pattern of correlation indicated a close relationship between eye-voice lead and several reading parameters associated with speed and accuracy. A large eye-voice lead was closely associated with faster and more accurate reading as well as fewer regressions and nearly absent silent pauses; conversely, a small eye-voice lead was associated with slower and more inaccurate reading, many pauses and regressions. Notably, it is the number of fixations, as well as pauses that carries this relationship, whereas the mean duration of fixations and pauses showed much weaker or non-significant relationships. Finally, utterance duration was also related to the eye-voice lead; this may indicate that, however, small, changes in utterance duration mark individual differences associated with the overall ability to integrate the various sub-components of reading behavior.

Due to limitations in sample sizes, correlations were examined in the whole sample of children. In view of the general differences in performance between the two groups, this might have been expected to inflate the size of the correlations. Examining the pattern of correlation within each of the groups of children yielded quantitatively very similar results; however, it still remains to be verified in larger samples of dyslexic and control readers whether the pattern of correlations found separately holds for these two groups.

## Discussion

In this study, we jointly measured several voice and eye movement parameters. Thus, the results allow us to make a comprehensive description of the reading profile of dyslexic and control readers. In reading texts, dyslexic children were slower than control peers. This slowness was expressed in a large number of silent pauses and sounding-out behaviors as well as slightly longer word articulation times. In scanning the text, their eyes were ahead of their voice, but much less so than in skilled readers, indicating that word processing and utterance production were much closer in time than in skilled readers. Errors such as word substitutions and non-word productions were few but considerably more than in control readers, whose accuracy was nearly flawless.

In comparing group differences across different parameters, standard statistical tests were not highly informative. Indeed, as expected, dyslexic children were different from control readers in nearly all parameters. A shortcoming of standard parametric analyses is that they do not easily cope with the over-additivity effect that is present in comparisons across groups, which vary for some general processing ability (Faust et al., 1999); namely, independent of their specific task characteristics, more difficult conditions tend to produce greater group differences. Indeed, previous research on dyslexia has underscored the tendency toward proportional differences between dyslexic and control readers across a large spectrum of conditions and tasks (e.g., Paizi et al., 2011). This has been interpreted as due to a multiplicative interaction between large basic differences in graphemic processing and condition difficulty (De Luca et al., 2010; Zoccolotti et al., 2008). Therefore, the possibility of comparing the reading sub-components/parameters by using a dimensionless measure such as the ratio, which provides information on the proportional size of the effect, seems more interesting.

### Ratios in the comparison of group performance

Across several eye movement and voice parameters, performance of dyslexic and control readers was expressed by a ratio of about 2. This was the case for total reading aloud time, total viewing time (whether aloud or silent) and number of fixations, as well as reading time on the MT test. Thus, we can consider a factor of 2.0 as a reference value to evaluate the size of group differences in reading parameters. The presence of consistent proportional differences across various different parameters is coherent with the literature, which indicates that dyslexic readers show a consistent deficit across very different stimulus materials (such as short vs. long words or words vs. non-words) when analyzed in ways that allow detecting global components in the data (e.g., Zoccolotti et al., 2008; Van den Broeck and Geudens, 2012).

Thus, within this perspective it is possible to consider unitarily group differences that, if expressed in terms of absolute scores, seem to be different in size. Consider the case of viewing time during silent or aloud reading. In keeping with Anderson and Anderson and Swanson's (1937) early observations, group differences were numerically greater in aloud than in silent reading; but the ratios for these two conditions were very similar, indicating that the increase in group differences can be parsimoniously explained as due to the greater difficulty in the aloud (than the silent) condition, without the need to refer to additional specific effects in the reading aloud condition. Furthermore, the 2-ratio approach allowed us to detect parameters that showed effects which were proportionally smaller or greater than this reference. In particular, pronunciation times showed only a 1.2 ratio or 20% prolongation; by contrast, number of pauses showed a much greater effect with a 4.5 ratio. Thus, the slow reading of dyslexic readers might be characterized as related more to an increase in the frequency of silent pauses and less (although significantly) to slowed articulation times. The data on pronunciation times also underscore the limits of using traditional standardized measures of effect size (such as Cohen's *d*). When *d* values were considered, group differences actually revealed a large group effect because of the very low inter-individual variability in pronunciation times. In this particular case, group differences in inter-individual variability were not merely due to differences in measurement reliability; rather, they reflect the well-known difference between cognitive and motor measures, with the former yielding much greater individual differences than the latter (e.g., Myerson et al., 2003).

However, some limits of the perspective based on ratio comparisons must also be underscored. Compared to models that make explicit predictions to test global components in the data (such as the *rate and amount model*, Faust et al., 1999; the *difference engine model*, Myerson et al., 2003; or the *state trace model*, Van den Broeck and Geudens, 2012), the use of ratios might be considered a rather rough measure for estimating the size of group differences in situations where group differences should be expected to increase multiplicatively due to the interaction of task difficulty and basic group differences in information processing. In particular, by teasing out different components of reaction time measures, models such as the difference engine model (Myerson et al., 2003) allow separating non-decisional from decisional components of the response. A similar outcome is reached with the diffusion model (e.g., Zeguers et al., 2011), which is based on very different assumptions. By contrast, referring to ratios does not allow distinguishing whether (and to what extent) group differences depend on decisional or non-decisional components of the response. At the same time, note that although the quoted models are considerably more powerful by themselves, they are typically used to deal with conditions that have several constraints and are usable only for rather specific predictions.^1^ Overall, there are clear limitations in examining ratios compared to more formal approaches to modeling group data in cognitive tasks. At the same time, this approach does provide a general profile of the group differences across reading parameters, which would be impossible to tackle using the quoted models.

Some caution should be taken to avoid over-interpreting differences in ratios across conditions/parameters. In particular, we feel this applies to the conditions which greatly deviate from the pattern of variability expected in the case of an over-additivity effect, i.e., appreciably larger SDs for the impaired (dyslexic) group than the control group. For example, the present data indicated very large differences in accuracy between the two groups; namely, dyslexic readers made 5.5 more errors than control readers in reading the text (and, based on data in Table 1, a similar 4.8 ratio was present for errors in the case of the MT standard reading test). However, on the basis of these high ratios it seems farfetched to consider that accuracy is more involved in the dyslexic deficit than speed. Indeed, skilled readers made an extremely small number of errors (or no errors), which is typical in transparent orthographies. We propose that this group difference can be explained in terms of a floor effect, i.e., several children did not actually make any substitution errors or engage in sounding-out behavior. This was clearly more frequent in the control group but spared performance in some parameters was present also in several dyslexic children. Accordingly, the large variability stems from the fact that only some of the children contributed to these measures, while some were flawless. Therefore, values for dyslexic children can easily override control's performance and generate very high ratios that cannot be directly compared with those based on time measures (where all individuals contribute some variability in generating the group average). Overall, it is difficult to directly compare accuracy with measures of eye movements or reading rate. However, in the accuracy measures, ratios may indicate that sounding-out behavior accounted for greater differences than word substitution errors. This finding is in keeping with previous observations in Italian children (Trenta et al., 2013) and indicates the tendency to phonologically recode words when a holistic approach to the target fails. In particular, Trenta et al. (2013) reported that sounding-out behavior was a significant predictor of dyslexic grouping in both text passages and word lists. Within the context of the present study, it is intriguing to observe that the “central” nature of sounding-out (called hesitations) was probably first understood by Fairbanks (1937), although he has not yet been credited for it. Interestingly, he noted that “*substantial positive inter-correlations between errors, hesitations, regressions … were obtained, and the coefficients almost always were greater in poor reading … Inclusion of hesitations as errors raised the correlation between errors and regressions, suggesting that even though the determination of hesitations is a qualitative step, more central errors were included.*”

### Eye-voice lead

The present study aimed to investigate eye-voice lead. In the 1930s, this phenomenon was the center of attention of a group of studies on eye movements in reading. But when research began to focus on examining silent reading conditions, interest in the eye-voice lead reduced considerably.

The results of the present study confirm a clear difference in eye-voice lead in dyslexic and matched skilled readers. This finding, which was obtained with children who speak a language with regular orthography, is in keeping with the early observations of Buswell (1921) and Fairbanks (1937). At that time, this measure created a considerable technical challenge and investigations could be carried out only in selected laboratories. Contemporary standard eye movement equipment and appropriate audio software have simplified the recording of eye-voice lead; indeed, this measure can now be more easily included in experimental studies on eye movements in dyslexia.

Eye-voice lead is very sensitive to local influences (Fairbanks, 1937; Inhoff et al., 2011) and varies systematically along the line of text and as a function of psycholinguistic parameters of the stimuli, such as high vs. low frequency words. It also decreases when a reader needs to regress to a previous word. At the same time, the present results indicate that the systematic individual tendency can be detected by analyses that cut across these locally determined variations. Some individuals tend to move their eye scanning forward “without waiting” for the execution of voice output, whereas others are so slow in decoding written words that the voice output flow remains nearly simultaneous with decoding. In this sense, eye-voice lead probably represents the idiosyncratic reading style of an individual more than any other parameter. In this view, after years of reading experience the reader develops a systematic tendency to trade-off visual and vocal processing. Within the perspective of the present study, it is interesting that variations in eye-voice lead showed a ratio in the comparison between dyslexic and control readers in line with the reference 2-ratio (i.e., 1.7 for both types of obtained estimates). This indicates that eye-voice lead reflects the proportional differences between the two groups.

Interestingly, the pattern of correlation indicates a widespread relationship between eye-voice lead and critical parameters such as reading aloud time, number (and total duration) of pauses, total viewing time and percentage or regressions. Notably, a significant correlation was present also for utterance duration. Although variations in utterance durations were comparatively small, they contributed quantitatively to participants' setting-up style for integrating voice and eye parameters. Importantly, the eye-voice lead in aloud reading was also highly correlated with total viewing time in silent reading; indeed, the size of this correlation was indistinguishable from those described above. Since eye-voice lead and viewing time in silent reading were evaluated on a different text, this is in line with the idea that eye-voice lead represents a stable individual trait.

The idea that readers develop an idiosyncratic style of reading is in keeping with observations by Carver (1982, 1992). He proposed that through prolonged practice readers develop an optimal reading rate, which is best suited to comprehend the thoughts contained in sentences (which corresponds to ca. 300 words per minute in college students). Carver (1982) referred to this as a “rauding” rate to mark the continuity between reading and listening (or auding). Therefore, in this view the optimal reading rate is that used by an individual to optimize reading comprehension. In keeping with this idea, Carver (1982) observed lower comprehension efficiency when readers were forced to read at a faster or slower rate than their optimal one.

### Decomposing dyslexics' reading slowness

One interesting question is how reading slowness in dyslexic readers expresses in the various sub-components of the reading task.

Dyslexic readers presented a much larger number of silent pauses than control readers. Indeed, in many cases the reading of skilled readers flowed continuously, with effective co-articulation of subsequent sounds regardless of whether or not they were part of the same word. It is clear that during silent pauses eye scanning is occurring through numerous fixations of the as yet unpronounced word, contributing to reading slowness by increasing the number of fixations. Furthermore, dyslexic children engaged much more often in other time- consuming behaviors such as re-sounding the stimulus target before uttering it correctly. Note that some lengthening was also detected in the articulation times of words pronounced without detectable hesitations or silent pauses.

Although the literature on reaction times to single-word presentation is immense, only a handful of studies have examined articulation times to singly presented words (Davies et al., 2013; Martelli et al., 2013). Both of the latter studies found that pronunciation times varied as a function of the lexicality and length of the stimulus (and also frequency in the case of Davies et al., 2013). These findings indicate that coding processes may indeed continue after response onset. Consistent results were also reported by Balota and Abrams (1995). These authors found that when the same arbitrary articulatory response was requested in a lexical decision task the duration of the utterance varied for stimuli of different word frequency. These findings, which indicate a spillover from the decoding phase of processing to the execution phase, point to a continuity between reaction and articulation times and speak against a clear-cut separation between cognitive and motor times. Notably, the effect of variables such as lexicality (Davies et al., 2013; Martelli et al., 2013) or frequency (Davies et al., 2013) on articulation times was very small in the case of singly presented targets; however, it was significant thanks to the extremely small variances present in the case of articulation times. For example, Martelli et al. (2013) found a delay of 6% (or a ratio of 1.06) between the articulation times of dyslexic and control readers across words and non-words. The effect was only 3% (or a ratio of 1.03) in the case of words and 9% (or a ratio of 1.09) in the case of non-words. Compared with these figures, the presence of a 24% delay (or ratio of 1.24) observed here in reading a meaningful text indicates an appreciable increase in the slowing in pronunciation in the case of multiple as opposed to single words (3%), even though the effect (24%) remains quantitatively small. In keeping with the idea that articulation times may indeed capture some of the variance connected with decoding and comprehension processes, it is interesting that they were correlated with a holistic parameter such as the eye-voice lead, that is, individuals with a smaller eye-voice lead showed longer articulation times than individuals with a larger eye-voice lead.

To the best of our knowledge, pronunciation times during reading a text passage have never been reported in dyslexic children; therefore, a comparison with other reading studies is impossible. However, the problem of comparing the predictive value of pauses vs. articulation times has received some attention in research on the RAN paradigm. Several studies have been based on the hypothesis that pause time is responsible for the well-known correlation between RAN tasks and reading speed (Neuhaus et al., 2001; Neuhaus and Swank, 2002; Georgiou et al., 2006). However, when pauses and articulation times were simultaneously recorded, both were highly correlated with reading speed and accuracy, at least for Greek and English observers (Georgiou et al., 2008). Indeed, Georgiou et al. (2008) proposed that the variance which is common to pause and articulation times is most correlated with reading.

Overall, these findings indicate that the slowing of dyslexic readers expresses through a variety of modifications in different parameters even though they occur within very different scales, i.e., producing large, evident differences as in the case of silent pauses (and number of fixations) or small, more difficult to detect, differences as in the case of articulation times.

### Toward an interpretation of the developmental reading deficit

The present results are in keeping with the general idea that reading requires the efficiency of several cognitive processes, including fast and effective visual scanning, visual selective attention, fast retrieval of lexical entries, short-term memory, and executive functioning. The question is whether it is possible to have a unitary interpretation of the deficit of dyslexic children in dealing with the multiple components of reading or whether separate interpretations are necessary.

Several interpretations have focussed on the efficiency of each of these processes. For example, it has been proposed that dyslexic children show impaired visual scanning (e.g., Vidyasagar and Pammer, 1999). Others studies attributed deficient reading to a reduced visual-attention span (Prado et al., 2007) or to multisensory spatial attention deficits (Facoetti et al., 2010). And other authors have reported that dyslexic readers have low verbal short-term memory (e.g., Wagner and Torgesen, 1987). It has also been reported that developmental dyslexia is associated with poor executive functioning (e.g., Brosnan et al., 2002). This is just a brief list of the complex pattern of impairments which can putatively cause or contribute to causing developmental dyslexia. In this respect, it must be kept in mind that increasing evidence indicates that developmental disorders present a large spectrum of homotopic and heterotopic co-morbidities, which make causal interpretations problematic (for reviews see Pennington, 2006; Pennington and Bishop, 2009). Thus, we think that at least some of the quoted findings represent individual associations, not causal relationships.

An alternative, more parsimonious, interpretation is that dyslexic children have a single predominant deficit; but, given the interwoven nature of the reading task, this deficit spreads to affect all sub-components of reading behavior. Based on previous evidence, the most likely candidate for this nuclear deficit seems to be impaired word decoding. This deficit is very clear in experimentally isolated conditions (e.g., in vocal RTs to singly presented targets). When, in more natural conditions, the child has to couple this impaired decoding with the scanning of visual orthographic stimuli, holding the output phonological traces in memory up to utterance production etc., the impairment increases proportionally simply because of the difficulty in integrating a defective performance into a complex task. In this view, other impairments in relevant processes (such as visual scanning or short-term memory) may co-occur and exacerbate the reading pattern but they do not necessarily account for a severe and selective deficit in dealing with the multiple task requirements intrinsic in the reading task.

Nevertheless, even if one accepts this interpretation, the question is still open concerning the most likely mechanism at the base of the impairment in basic word decoding. Notably, the present results do not offer direct information on this point and only speculative interpretations can be presented. Based on a large body of literature, we feel that the two most likely candidates for this impairment are a deficit in visual or phonological processes. In the first class of mechanisms, one finds deficits in magnocellular functioning (Stein and Walsh, 1997) or visual crowding (Spinelli et al., 2002). For example, in visual crowding word recognition is possible only within an uncrowded window (Pelli et al., 2007), and differences in the size of this window account for the differential efficiency in the reading of dyslexic and control readers (Martelli et al., 2009). In the second class of mechanisms are hypotheses of continuity between acoustic deficits and phonological coding (Goswami et al., 2011). Both children and adults with developmental dyslexia have deficits in perceiving syllable stress in speech (Leong et al., 2011), and dyslexic children have difficulty in discriminating rise times of the speech signal for auditory presented syllables (Goswami et al., 2011). Poor sensitivity to the rhythmic structure of speech produces “*consequences for developing the high-quality phonological representations of spoken words necessary for the acquisition of literacy*” (Goswami, 2011). In particular, according to the temporal sampling framework, children with dyslexia have problems at the temporal integration window, which is typical of the syllable analysis, i.e., around 200 ms, not in the 20–50 ms range, which is typical of phonemes (Goswami, 2011). Although critical experiments on this model have been carried out in the acoustic modality (e.g., Goswami et al., 2011), we thought it would be interesting to compare the predictions of the model concerning the timing of information acquisition in the visual modality. To this aim, the most critical parameter seems to be the duration of individual fixations during reading. In the present study, the peak of fixation duration distribution (time window 175–225 ms) was comparable in young skilled and dyslexic readers; however, the frequency was higher in dyslexic children in the same temporal window than in other time windows, whereas the frequency of very short fixations was nearly comparable. Thus, it seems that there was a visual oversampling at a time window corresponding to the one that is critical for phonological syllable analysis. Based on these preliminary observations, it would be interesting to investigate the temporal sampling framework within the visual modality.

## Conclusions

Overall, the results of the present study allow for a comprehensive description of the reading profile of dyslexic and control readers during the reading of a text, including several eye and voice parameters and accuracy measures. In this context, the eye-voice lead represents a key phenomenon that describes well the complexity of the reading task. Research on the eye-voice lead was at the center of attention in the early part of twentieth century, but then lost impetus; it seems that this measure (which is considerably easier to gather with modern equipment) might provide interesting information about reading efficiency.

Across several parameters, the difference in performance between dyslexic and control readers was expressed by a ratio of about 2. A much lower ratio was measured for pronunciation parameters, indicating that this subcomponent weighed less than other subcomponents in the overall reading time. Referring to proportional differences allows for a more parsimonious interpretation of the reading deficit; in particular, we propose that an impairment in word decoding is the key deficit and that it spreads in such a way as to produce severe difficulty in dealing with the multiple task requirements intrinsic to reading.

### Conflict of interest statement

The authors declare that the research was conducted in the absence of any commercial or financial relationships that could be construed as a potential conflict of interest.

## Footnotes

^1^For example, the rate and amount (Faust et al., 1999) as well as the difference engine (Myerson et al., 2003) model are suited to account for group differences in performance on timed tasks within a relatively short time range (ca. 0–3 s); further, they are based on rather strict linear relationships in the data and cannot deal with closed scale data (such as accuracy measures). Accordingly, although they are convenient for studying RTs, their extension to more natural task conditions, such as reading a meaningful text, is comparatively difficult. A model such as the state trace model (e.g., Van den Broeck and Geudens, 2012) does not have some of these limitations (particularly the requirement of linear predictions or the restriction to closed scales) but still applies best to selected experimental conditions; furthermore, by requiring an overlap in performance between the two groups, it is best suited for experiments with ad-hoc designs, which allow producing such an overlap. Similar considerations apply to the diffusion model. This has the advantage of dealing jointly with accuracy and time data but also requires ad-hoc designs and is difficult to extend to more natural situations, such as those in the present study. Note that in the particular case of reading accuracy may be very high in natural conditions at least in skilled readers, thus violating one requirement of the model.
